# Low serum vitamin D-status, air pollution and obesity: A dangerous liaison

**DOI:** 10.1007/s11154-016-9388-6

**Published:** 2016-09-19

**Authors:** Luigi Barrea, Silvia Savastano, Carolina Di Somma, Maria Cristina Savanelli, Francesca Nappi, Lidia Albanese, Francesco Orio, Annamaria Colao

**Affiliations:** 1 I. O.S. & COLEMAN Srl, 80011 Acerra, Naples, Italy; 2 Dipartimento di Medicina Clinica e Chirurgia, Unit of Endocrinology, Federico II University Medical School of Naples, Via Sergio Pansini 5, 80131 Naples, Italy; 3 IRCCS SDN, Napoli Via Gianturco 113, 80143 Naples, Italy; 4 Department of Sports Science and Wellness, “Parthenope” University of Naples, Naples, Italy

**Keywords:** Environmental factor, Vitamin D, Air pollution, Obesity, Nutritionist

## Abstract

The aim of this review is to provide a general overview of the possible associations among the vitamin D status, air pollution and obesity. Sunlight exposure accounts in humans for more than 90 % of the production of vitamin D. Among emerging factors influencing sunlight-induced synthesis of vitamin D, prospective and observational studies proved that air pollution constitutes an independent risk factor in the pathogenesis of vitamin D hypovitaminosis. In addition, environmental pollutants can affect risk of obesity when inhaled, in combination with unhealthy diet and lifestyle. In turn, obesity is closely associated with a low vitamin D status and many possible mechanisms have been proposed to explain this association. The associations of air pollution with low vitamin D status on the hand and with obesity on the other hand, could provide a rationale for considering obesity as a further link between air pollution and low vitamin D status. In this respect, a vicious cycle could operate among low vitamin D status, air pollution, and obesity, with additive detrimental effects on cardio-metabolic risk in obese individuals. Besides vitamin D supplementation, nutrient combination, used to maximize the protective effects against air pollution, might also contribute to improve the vitamin D status by attenuating the “obesogen” effects of air pollution.

Sunlight exposure provides in humans for more than 90 % of the production of vitamin D, a liposoluble hormone that is known to exert a wide range of hormonal functions in skeletal and non-skeletal tissues [1]. In particular, solar ultraviolet (UV)-B radiation (UVB; wavelengths of 290 to 315 nm) stimulates the synthesis of vitamin D3 from 7-dehydrocholesterol in the epidermis of the skin [2]. Circulating vitamin D3 is transported to the liver, where it is hydroxylated to form 25-hydroxyvitamin D3 (25(OH)D) or calcidiol, the major circulating form of vitamin D. Thereafter, the 25-hydroxyvitamin D3–1-hydroxylase enzyme catalyzes a second hydroxylation of 25(OH)D in the kidneys, resulting in the formation of 1,25(OH)_2_D or calcitriol, the active form of vitamin D [2]. In this respect, vitamin D is actually more like classified as an active circulating pre-hormone than a vitamin, a substance that is obligatorily required from the diet. Hence, inadequate radiation or insufficient cutaneous absorption of solar UVB is one of the cardinal causes of vitamin D deficiency. The penetration of UVB photons into the skin is impaired by various factors, including season, time of day, geographical location skin pigmentation, sunscreen use, aging, and cultural background. However, different epidemiological studies suggest that worldwide prevalence of a low vitamin D status is higher than expected also in spite of an abundant sun exposure [3]. Among emerging factors influencing sunlight-induced synthesis of vitamin D, a great body of evidence indicates that environmental aerosol pollutants reduce the effectiveness of sun exposure in producing vitamin D in the skin by absorbing and scattering solar UVB radiation [2]. Thus, air pollution, determining the percentage of the ground level of solar UVB photons, may play a significant independent role in the development of vitamin D deficiency [4].

The aim of this review is to provide a general overview of the possible associations among the vitamin D status, air pollution and obesity.

## Air pollution and low vitamin D status

Exposure to chronic, low concentrations of air pollutants, generated as a consequence of urbanization, including automobile exhaust, heating buildings and particles generated from industrial plants, constitutes an environmental global risk to human health [4]. In general terms, air pollution is made up of gases and particulate matter (PM). Particulate matter is classified according to diameter into PM10 microns, PM2.5 μm, the major component of pollutant particles, primarily derived from direct emissions from combustion processes, and ultrafine particles [5]. It was estimate that nearly 80 % of the world’s population lives in areas where air pollution levels largely exceeding the limit values set by the air quality guidelines, established by the World Health Organization [6]. Air pollution, especially the tropospheric ozone, effectively absorbs UVB radiation, thereby reducing the quantity of photons reaching the earth’s surface. As the skin synthesis of vitamin D, which occurs through the action of sunlight, is not surprising that a reduced radiation caused by air pollution constitutes one of the main causes of vitamin D deficiency [4]. Accordingly, urban residents, especially in areas with high levels of ambient air pollution, tend to be less engaged in outdoor activities and have a higher prevalence of D hypovitaminosis compared to rural inhabitants [7].

Prospective and observational studies, conducted on populations living in different geographic areas, proved that air pollution constitutes an independent risk factor in the pathogenesis of vitamin D hypovitaminosis [4]. A cross-sectional study conducted in Belgium on postmenopausal women engaged in outdoor activities, showed that the tropospheric ozone increased the prevalence of a low vitamin D status [8]. In particular, in this study the serum levels of vitamin D and parathormone (PTH) of 47 women living in rural areas were compared with those of 38 women living in Brussels. As expected, in urban residents the prevalence of vitamin D insufficiency was significantly higher than rural residents (38 % vs 18 %), while the concentration of ground-level ozone was 3 times higher in urban areas than in rural areas. In a cross-sectional study conducted in urban and rural areas of Iran, Hosseinpanah et al. [9] included 200 healthy women living in two separate zones of approximately similar latitude, but with different levels of air pollution. The Authors found that the prevalence of hypovitaminosis D was higher in women living in the more polluted area compared those living in the less polluted area, confirming that air pollution plays a significant independent role in vitamin D deficiency. Baïz et al. [10] investigated the association between gestational exposure to ambient urban air pollution and levels of vitamin D in the cord blood in french mother-child pairs, and found that the pollutant exposure, especially during late pregnancy, contributed to lower vitamin D levels in offspring, thus affecting the child’s risk of developing diseases later in life. The association between air pollution and low vitamin D status was also investigated among children by Agarwal et al. [11], who compared serum 25(OH)D levels of 34 children aged between 9 and 24 months, residents in Mori Gate, an area of ​​Delhi known for the high levels of air pollution, with those of 34 children matched by sex and age, residents of Gurgaon, a less polluted area of ​​the city. Children living in the highly polluted area had average serum concentrations of 25(OH)D significantly lower by 54 % compared to those living in the less polluted area of the city. In addition, the quality of the air has been proven to retain an inverse association with 25(OH)D among children aged 4–10 years and living in Iran, a region with plenty of sunlight, but in a highly air-polluted city, such as Isfahan [12].

## Inhaled pollution and obesity

Aside from the mutagenicity and carcinogenicity of particulate air pollution from combustion source, the largest source contributing to the PM2.5 mass [13], epidemiological studies show that air pollution exposure is associated with adverse respiratory health effects [14]. Beyond the effects on respiratory system, current data strongly support that exposure to air pollutants may not only carry a perivascular and peribronchial inflammation, but can increase both systemic inflammation and oxidative stress, the main links of air pollution with cardiovascular diseases and obesity [15–17]. Mechanistically, inhaled pollution particles induce a local inflammatory response in the lung that is initiated by alveolar macrophages and airway epithelial cells. Endothelial dysfunction and reactive oxygen species generation via activation of alveolar macrophages and systemic vascular oxidases, including nicotinamide adenine dinucleotide phosphate (NADPH), mitochondrial and xanthine oxidases, appear to represent early steps in this pro-inflammatory status [18]. In particular, PM2.5 exposure enhanced the expression in alveolar macropahges of proinflammatory cytokines, such as interleukin-6 and tumour necrosis factor-α [5]. Subsequently, systemic mediators translocate from the lung into the circulation eliciting the classic systemic inflammatory response, with production of acute phase proteins by the liver [5]. In addition, air pollutants might act as “obesogens” by altering the methylation of peroxisome proliferator-activated receptor gamma (PPARγ) or PPARγ target molecules, known to exert a pivotal role in the regulation of adipogenesis [19–21], or via their binding to the α and β estrogen receptors (ER), actively involved in the regulation of energy metabolism pathways [22].

Besides the obesogen effect of tobacco smoking [23–25], an increasing number of associative studies have suggested that inhaled environmental pollutants, combined with unhealthy diet and lifestyle, are associated with a propensity to obesity, metabolic syndrome, and insulin resistance, and are able to contribute to chronic non transmissible diseases [26], including cardiovascular disease and type 2 diabetes mellitus, all conditions that are characterized by systemic inflammation, in both adults [27–29] and children [23]. In particular, the association between obesity and PM2.5 has been extensively evaluated by a meta-analysis including three large prospective cohort studies and 14 panel studies with short-term follow-up [30]. Results of this meta-analysis indicate that obese people may be more susceptible to the cardiovascular health effects of ambient PM2.5, also after adjusting for a number of potential confounding factors.

## Low vitamin D status and obesity

Obesity and vitamin D deficiency are among the most important modifiable risk factors for non-transmissible chronic diseases [31]. In particular, there is an inverse association of mortality risk and vitamin D levels, although this association could be indirectly mediated through obesity itself [32]. Indeed, obesity is closely associated with a low vitamin D status, as higher body mass index leads to lower vitamin D status [33]. Vitamin D receptors (VDR) are widely expressed in adipose and β-pancreatic cells, and both cells also possess also the capacity to activate in loco vitamin D by having the enzyme 25-hydroxyvitamin D 1-α-hydroxylase [34]. Through its receptors, Vitamin D exerts relevant effects *in vitro* on gene expression and proteins related to adipose tissue differentiation and metabolism. In particular, vitamin D inhibits the differentiation of pre-adipocytes, suppresses a number of transcriptional regulators and functional proteins exerting a key role in adipocyte metabolism, such as PPARγ, lipoprotein lipase, protein aP2, a carrier of fatty acids necessary for lipolysis, CCAAT/enhancer-binding protein (C/EBP) and sterol-regulatory element-binding protein-1 (SREBP-1) [32].

Although various epidemiological studies and clinical trials show that obese individuals have low circulating levels of 25(OH)D, with an inverse relationship between serum 25(OH)D and PTH levels, the relationship between obesity and 1,25(OH)_2_D, is less clear [32]. Under conditions of low vitamin D status, low serum 25(OH)D levels tend to be associated with high serum PTH levels. In turn, PTH stimulates the production of 1,25(OH)_2_D, which has a number of extra-skeletal effects, including on adipose tissue [35]. The 1,25(OH)_2_D exerts different effects on adipose tissue, as it stimulates the transcription of adipogenic factors and reciprocally inhibits lipolysis by binding to the VDRs expressed on adipocytes [36]. In addition, 1,25(OH)_2_D modulates the chronic inflammation in adipose tissue by reducing the proinflammatory cytokines secreted from adipose tissue [35].

Many possible mechanisms might account for the low vitamin D status during obesity, including lower dietary intake of vitamin D by obese individuals [31], lesser exposure of skin to sunlight in obese individuals, due to less outdoor activity than leaner individual [31], decreased intestinal absorption due to malabsorptive bariatric procedures, impaired 25-hydroxylation and 1-α hydroxylation in adipose tissue. Perhaps, the most likely explanation for low vitamin D status in obesity is that, due to its lipophilicity, vitamin D is largely stored in the adipose tissue [37]. Nevertheless, a volumetric dilution related to the greater volume of distribution of 25(OH)D in tissue mass in obese individuals could be considered as a reasonable cause of the low vitamin D status [38]. Finally, it has also been suggested that accrual of adipose tissue obesity could result from an excessive adaptive “winter response”, and that the decline in vitamin D skin synthesis, due to reduced sunlight exposure, contributes to the tendency to increase fat mass during the colder periods of the year [39]. Thus, the increased storage capacity for vitamin D in obese individuals is likely to reduce the circulating 25(OH)D concentrations [31]. Accordingly, serum levels of vitamin D showed a relatively smaller increase in obese subjects as compared with that in non-obese subjects after either 24 h of UVB radiation or oral vitamin D supplementation [40]. On the other hand, the possibility can be envisaged that low vitamin D itself could contribute to obesity or reduce weight loss [32]. A low vitamin D status is known to induce secondary hyperparathyroidism that increases the intracellular levels of ionic calcium in adipocytes [41]. Increased intracellular calcium in adipocytes can increase the expression of fatty acid synthase, a key regulatory enzyme in the deposition of lipids, and decrease lipolysis [42].

Although the direction of the association between low vitamin D and obesity still remains debatable [43], a number of clinical and experimental studies have provided evidence for the role of obesity as a causal risk factor for the development of vitamin D deficiency [33]. In particular, it has been calculated that each unit increase of BMI was associated with a 1.15 % decrease of 25(OH)D [31]. In addition, it has been described a strong inverse association between vitamin D status with both subcutaneous and visceral adiposity [44]. Thus, low vitamin D can cause the adipose tissue accrual and compromise normal metabolic functioning, contributing to the adverse health effects associated co-morbidities, including insulin resistance and type 2 diabetes [45]. Experimental data have shown that large doses of vitamin D2 lead to increases in energy expenditure due to uncoupling of oxidative phosphorylation in adipose tissues [46]. However, clinical trials investigating the effects of increased in vitamin D status on body weight have not provided consistent data on possible effects of vitamin D supplementation on weight loss [47, 48]. In this context, it has been reported that normalization of 25(OH)D levels in vitamin D-insufficient subjects with vitamin D supplementation could participate to prevent weight gain by reducing the 1,25(OH)2D production, likely through lowering PTH levels [49].

## Nutrition solutions: role of nutritionist

Taking into account the associations among vitamin D status, air pollution and obesity, some practical nutritional considerations could be drawn.

Despite that the anti-obesity effects of vitamin D supplementation is still matter of debate, the link between air pollution and obesity strongly support the use of adequate vitamin D supplementation in obese individuals according to the current guidelines, in association with a healthy diet and lifestyle, especially in those living in urban areas with high air pollution [4, 50]. The amount of vitamin D produced by an adult who is exposed to the sun to one minimal erythemal dose of UV radiation is equivalent to ingesting between 10,000 and 25,000 IU of vitamin D [51]. The Endocrine Society suggests 1000 IU/d for infants up to 6 months, 1500 IU/d for infants from 6 months to 1 year, at least 2500 IU/d for children aged 1–3 years, 3000 IU/d for children aged 4–8 years, and 4000 IU/d for everyone over 8 years. Higher levels of 2000 IU/d for children 0–1 year, 4000 IU/d for children 1–18 years, and 10,000 IU/d for children and adults 19 years and older may be needed to correct vitamin D deficiency [50]. Indeed, few foods contain vitamin D and clinical studies suggest that we may need more vitamin D than presently recommended to prevent chronic non-transmissible diseases [52, 53]. In particular, vitamin D is present in oil-rich fish, sunlight-exposed mushrooms, eggs, and milk [44]. Cod liver oil is a rich natural source of vitamin D; nevertheless, there is concern regarding its use at high doses due to its vitamin A content and possible contamination by heavy metals, such as mercury [54, 55]. Fortification with vitamin D of food, such as milk, bread and margarine, is associated with statistically significant improvements in serum 25(OH)D [56, 57]. However, fortification with vitamin D varies worldwide and the vitamin D content in the foods is quite variable.

Besides vitamin D supplementation, an improvement in the vitamin D status could be obtained also through the attainment of a healthy dietary pattern and weight loss. In this respect, it is well known that nutrients with higher antioxidant activity are recommended to reduce the risk of developing overweight/obesity, including as ω-3 polyunsaturated fatty acids (PUFA), monounsaturated fatty acids (MUFA), vitamins of complex B, vitamin C, vitamin E, in combination with a healthy diet regimen rich in fruits, vegetables, fibre, with a reduced intake of saturated fats, simple carbohydrates, and sweetened drinks. More recently, a number of clinical trials indicate that some nutrients can attenuate the oxidative damage induced by air pollution [58, 59]. In particular, Tong et al. in a randomized, double-blinded, controlled study, showed in healthy middle-aged adults the efficacy of 3 g/day the fish oil supplements provided protection against the adverse cardiac and lipid effects associated with air pollution exposure [58]. However, a recent study indicates that ω-3 PUFA nutritional supplements should be analysed for oxidation safety [60]. The combined supplementation with 800 mg/d of vitamin E and 500 mg/d of vitamin C showed an additive protective effect of both vitamins to the oxidative stress associated with air pollution, where vitamin E acts as antioxidant and vitamin C regenerates its oxidized form [59]. In addition, Riedl et al. [61] reported that broccoli extracts or sulforaphane, found in cruciferous vegetables such as broccoli and brussel sprouts, can have protective effects against air pollution via nuclear factor E2-related factor 2, the transcription factor responsible for the expression of antioxidant response element, the regulator of the endogenous antioxidant enzyme system [62]. Accordingly, in a randomized, placebo-controlled study, dietary intervention with broccoli sprouts enhanced the detoxification of some airborne pollutants, providing a frugal means to attenuate their associated long-term health risks [63].

## The dangerous liason among air pollution, obesity, and vitamin D

In this complex scenario, the well documented associations of air pollution with obesity on the one side, and with low vitamin D status on the other side, could provide a rationale for considering the obesity a further link between air pollution and low vitamin D status. In this respect, a vicious cycle could be envisaged, whereby on the one side the air pollutants, absorbing solar UVB radiation, reduce the effectiveness of sun exposure in producing vitamin D in the skin; on the other side, air pollutants, combined with unhealthy diet and lifestyle, might contribute to obesity as environmental “obesogens”. Obesity, through different mechanisms, including low dietary intake of vitamin D, less exposure of skin to sunlight and sequestration of vitamin D in the adipose tissue, might further worsen the low vitamin D status, thus increasing the cardio-metabolic risk in obese individuals, in a situation similar to the “winter response”. In turn, the low vitamin D status, through the secondary hyperparathyroidism and the increased the intracellular calcium in adipocytes, might increase adipogenic pathways, thus favouring weight gain and obesity. Accordingly, a number of studies reported that children’s air pollution exposures in the Mexico City Metropolitan Area was associated with systemic inflammation, endothelial dysfunction [64], altered appetite-regulating peptides, high risk of insulin resistance, obesity, type 2 diabetes, premature cardiovascular disease [65], early hallmarks of Alzheimer’s disease [66], and vitamin D deficiency [66, 67].

**Fig. 1:**
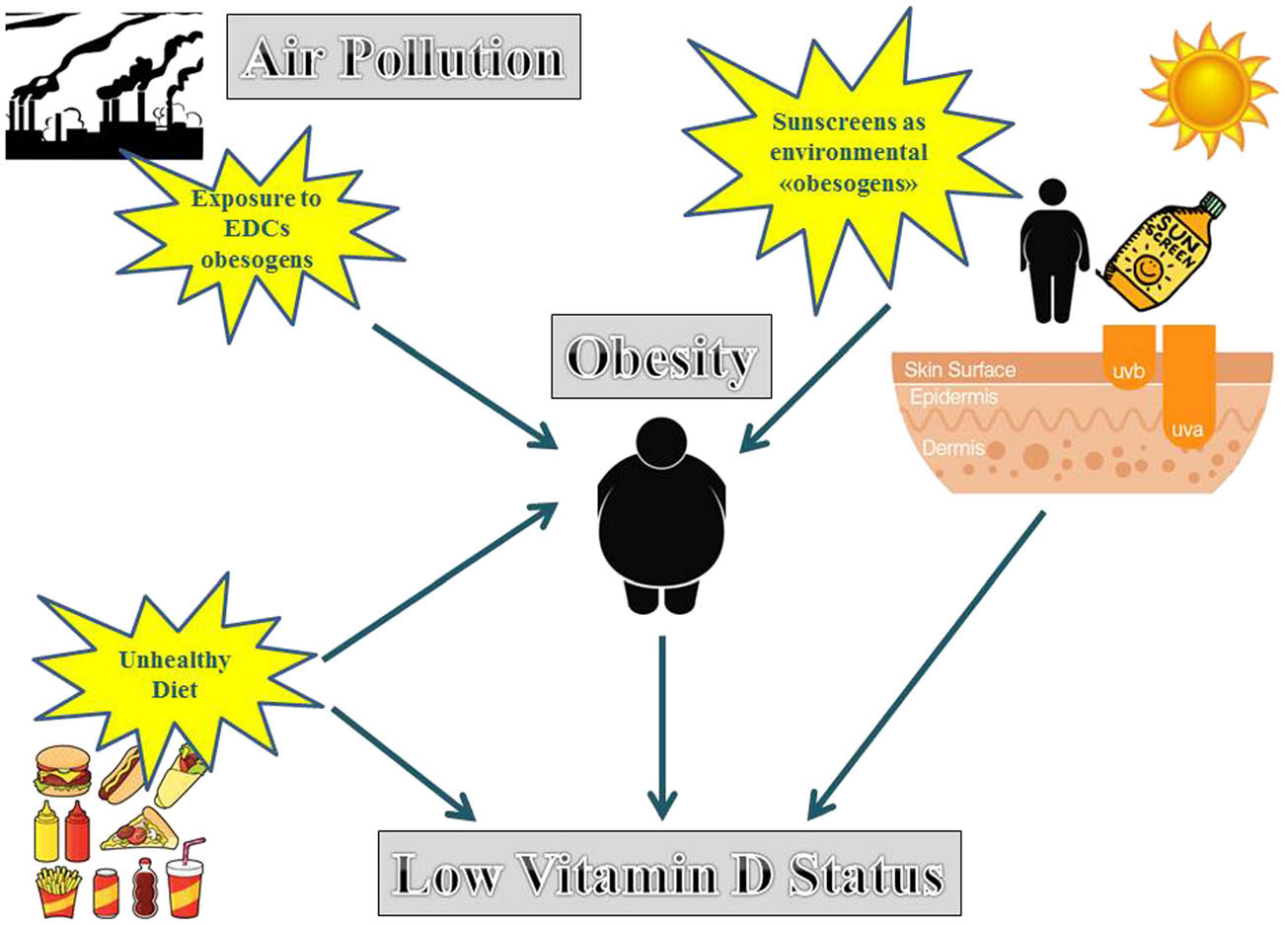
An intriguing hypothesis can be proposed, that, combined with unhealthy diet and lifestyle, the inadequately wearing of some sunscreens might cause a low vitamin D status either directly, by reducing the absorption of solar UVB radiation, and also indirectly, by inducing obesity, possibly by acting as environmental “obesogens”. Sequestration of vitamin D in the adipose tissue further worsens the low vitamin D status and increases the metabolic risk in obese individuals

The use of sunscreens might provide a novel aspect of associative link between obesity, pollution and low vitamin D status. Although still debatable [68–70], the regular use of sunscreens in sufficient amounts exerts protective effects on sunburn and might be essential for prevention of skin cancers, UV-induced immunosuppression, and photoaging. Although it has been reported that wearing a sunscreen with a sun protection factor of 30 reduces vitamin D synthesis in the skin by more than 95 %, a proper and adequate use of sunscreens is unlikely to affect vitamin D status adversely, as a sufficient amount of UVB radiation would reach the skin surface for production of vitamin D. Accordingly, markers for vitamin D deficiency, including 25(OH)D and PTH, are generally not affected due to use of sunscreens in Brazil, one of the countries of the world with greater extent of land in proximity to the sun [71, 72]. In addition, the adequate use of sunscreens contributes to promoting healthier lifestyle options that include outdoor physical activity. Nevertheless, to achieve the desirable level of protection, sun lotions and creams consist of cocktail of chemical filters. Percutaneous absorption and endocrine disrupting activity of small-sized organic and nano-sized inorganic UV filters have been reported [73]. It is well known that a number of endocrine disruptors (EDCs) can act as “obesogens” by promoting adipogenesis, intra-adipocyte lipid accumulation, and insulin resistance through different regulatory pathways. These regulatory pathways include the PPAR-γ pathways and the agonistic estrogenic effects via ER α and β, the main targets of EDCs involved in adipose tissue and energy metabolism [22, 74]. Although there is no evidence in the literature of a direct involvement of sunscreens as environmental “obesogens”, a role as EDCs in the context of their ability to induce reproductive disorders by acting as estrogenic compounds has been highlighted in a recently published review [75]. Hence, an intriguing hypothesis can be proposed, that an inadequately wearing of some sunscreens might cause a low vitamin D status not only directly by reducing the absorption of solar UVB radiation, but also indirectly acting as “obesogen” EDCs, combined with unhealthy diet and lifestyle. Sequestration of vitamin D in the adipose tissue further worsens the low vitamin D status and increases the cardio-metabolic risk in obese individuals. The proposed vicious cycle acting between vitamin D, air pollution, and obesity, combined with unhealthy diet and lifestyle and with the possible contribution of the inadequate use of particular sunscreens acting as endocrine disruptors, is presented in Fig. 1.

## Conclusions

The associations of air pollution with low vitamin D status on the one side, and with obesity on the other side, could provide a rationale for considering obesity as a further link between air pollution and low vitamin D status, with the possible dual contribution of sunscreens either by reducing the skin production of vitamin D, and, possibly, by acting as environmental “obesogens”. Vitamin D supplementation and nutrient combinations might be recommended to contribute to the protective effects against air pollution and to improve the vitamin D status by attenuating the “obesogen” effects of air pollution. A vicious cycle could be proposed among low vitamin D status, air pollution, and obesity, with additive detrimental effects on cardio-metabolic risk in obese individuals. Further prospective studies are needed to support the potential causal associations between vitamin D levels, air pollutants and obesity, and the impact in clinical practice of nutritional interventions to reduce the detrimental effects of the exposure to air pollutants and to improve the vitamin D status along with weight loss.

EDCs, endocrine disruptors; PM, particulate matter; PTH, parathormone; UV, ultraviolet; VDR, vitamin D receptors.

## References

[1] Webb AR, Holick MF (1988). The role of sunlight in the cutaneous production of vitamin D3. Annu Rev Nutr.

[2] Wacker M, Holick MF (2013). Sunlight and vitamin D: a global perspective for health. Dermatoendocrinol.

[3] Binkley N, Novotny R, Krueger D, Kawahara T, Daida YG, Lensmeyer G, Hollis BW, Drezner MK (2007). Low vitamin D status despite abundant sun exposure. J Clin Endocrinol Metab.

[4] Kurylowicz A. Impact of Air Pollution on Vitamin D Status and Related Health Consequences. Impact of Air Pollution on Vitamin D Status and Related Health Consequences, The Impact of Air Pollution on Health, Economy, Environment and Agricultural Sources, Dr. Mohamed Khallaf (Ed.), InTech. 2011; doi:10.5772/17838.

[5] Hiraiwa K, van Eeden SF (2013). Contribution of lung macrophages to the inflammatory responses induced by exposure to air pollutants. Mediat Inflamm.

[6] van Donkelaar A, Martin RV, Brauer M, Kahn R, Levy R, Verduzco C, Villeneuve PJ (2010). Global estimates of ambient fine particulate matter concentrations from satellite-based aerosol optical depth: development and application. Environ Health Perspect.

[7] Bailey BA, Manning T, Peiris AN (2012). The impact of living in rural and urban areas: vitamin D and medical costs in veterans. J Rural Health.

[8] Manicourt DH, Devogelaer JP (2008). Urban tropospheric ozone increases the prevalence of vitamin D deficiency among Belgian postmenopausal women with outdoor activities during summer. J Clin Endocrinol Metab.

[9] Hosseinpanah F, Pour SH, Heibatollahi M, Moghbel N, Asefzade S, Azizi F (2010). The effects of air pollution on vitamin D status in healthy women: a cross sectional study. BMC Public Health.

[10] Baïz N, Dargent-Molina P, Wark JD, Souberbielle JC, Slama R, Annesi-Maesano I, Mother-Child Cohort EDEN (2012). Study group. Gestational exposure to urban air pollution related to a decrease in cord blood vitamin d levels. J Clin Endocrinol Metab.

[11] Agarwal KS, Mughal MZ, Upadhyay P, Berry JL, Mawer EB, Puliyel JM (2002). The impact of atmospheric pollution on vitamin D status of infants and toddlers in Delhi, India. Arch Dis Child.

[12] Kelishadi R, Moeini R, Poursafa P, Farajian S, Yousefy H, Okhovat-Souraki AA (2014). Independent association between air pollutants and vitamin D deficiency in young children in Isfahan. Iran Paediatr Int Child Health.

[13] Lewtas J (2007). Air pollution combustion emissions: characterization of causative agents and mechanisms associated with cancer, reproductive, and cardiovascular effects. Mutat Res.

[14] Falcon-Rodriguez CI, Osornio-Vargas AR, Sada-Ovalle I, Segura-Medina P (2016). Aeroparticles, composition, and lung diseases. Front Immunol.

[15] Hutcheson R, Rocic P (2012). The metabolic syndrome, oxidative stress, environment, and cardiovascular disease: the great exploration. Exp Diabetes Res.

[16] Cui Y, Sun Q, Liu Z (2016). Ambient particulate matter exposure and cardiovascular diseases: a focus on progenitor and stem cells. J Cell Mol Med.

[17] Brook RD, Sun Z, Brook JR, Zhao X, Ruan Y, Yan J, Mukherjee B, Rao X, Duan F, Sun L, Liang R, Lian H, Zhang S, Fang Q, Gu D, Sun Q, Fan Z, Rajagopalan S (2016). Extreme air pollution conditions adversely affect blood pressure and insulin resistance: the air pollution and cardiometabolic disease study. Hypertension.

[18] Xu X, Yavar Z, Verdin M, Ying Z, Mihai G, Kampfrath T, Wang A, Zhong M, Lippmann M, Chen LC, Rajagopalan S, Sun Q (2010). Effect of early particulate air pollution exposure on obesity in mice: role of p47phox. Arterioscler Thromb Vasc Biol.

[19] Janani C, Ranjitha Kumari BDPPAR (2015). gamma gene--a review. Diabetol Metab Syndr.

[20] Tyagi S, Gupta P, Saini AS, Kaushal C, Sharma S (2011). The peroxisome proliferator-activated receptor: a family of nuclear receptors role in various diseases. J Adv Pharm Technol Res.

[21] Nappi F, Barrea L, Di Somma C, Savanelli MC, Muscogiuri G, Orio F, Colao A, Savastano S (2016). Endocrine aspects of environmental “obesogen” pollutants. J. Environ. Res. Public Health.

[22] Chen JQ, Brown TR, Russo J (2009). Regulation of energy metabolism pathways by estrogens and estrogenic chemicals and potential implications in obesity associated with increased exposure to endocrine disruptors. Biochim Biophys Acta.

[23] Kim HW, Kam S, Lee DH (2014). Synergistic interaction between polycyclic aromatic hydrocarbons and environmental tobacco smoke on the risk of obesity in children and adolescents: the U.S. National Health and nutrition examination survey 2003-2008. Environ Res.

[24] Møller SE, Ajslev TA, Andersen CS, Dalgård C, Sørensen TI (2014). Risk of childhood overweight after exposure to tobacco smoking in prenatal and early postnatal life. PLoS One.

[25] Ortega Hinojosa AM, Davies MM, Jarjour S, Burnett RT, Mann JK, Hughes E, Balmes JR, Turner MC, Jerrett M (2014). Developing small-area predictions for smoking and obesity prevalence in the United States for use in environmental public health tracking. Environ Res.

[26] Lim SS, Vos T, Flaxman AD, Danaei G, Shibuya K, Adair-Rohani H, Amann M, Anderson HR, Andrews KG (2012). A comparative risk assessment of burden of disease and injury attributable to 67 risk factors and risk factor clusters in 21 regions, 1990-2010: a systematic analysis for the global burden of disease study 2010. Lancet.

[27] Weichenthal S, Hoppin JA, Reeves F (2014). Obesity and the cardiovascular health effects of fine particulate air pollution. Obesity (Silver Spring).

[28] Ponticiello BG, Capozzella A, Di Giorgio V, Casale T, Giubilati R, Tomei G, Tomei F, Rosati MV, Sancini A (2015). Overweight and urban pollution: preliminary results. Sci Total Environ.

[29] Wei Y, Zhang JJ, Li Z, Gow A, Chung KF, Hu M, Sun Z, Zeng L, Zhu T, Jia G, Li X, Duarte M, Tang X (2016). Chronic exposure to air pollution particles increases the risk of obesity and metabolic syndrome: findings from a natural experiment in Beijing. FASEB J.

[30] Dai L, Bind MA, Koutrakis P, Coull BA, Sparrow D, Vokonas PS, Schwartz JD (2016). Fine particles, genetic pathways, and markers of inflammation and endothelial dysfunction: analysis on particulate species and sources. J Expo Sci Environ Epidemiol.

[31] Vimaleswaran KS, Berry DJ, Lu C, Tikkanen E, Pilz S, Hiraki LT, Cooper JD, Dastani Z, Li R (2013). Causal relationship between obesity and vitamin D status: bi-directional Mendelian randomization analysis of multiple cohorts. PLoS Med.

[32] Earthman CP, Beckman LM, Masodkar K, Sibley SD (2012). The link between obesity and low circulating 25-hydroxyvitamin D concentrations: considerations and implications. Int J Obes.

[33] Prasad P, Kochhar A (2016). Interplay of vitamin D and metabolic syndrome: a review. Diabetol Metab Syndr.

[34] Cândido FG, Bressan J, Vitamin D (2014). Link between osteoporosis, obesity, and diabetes?. Int J Mol Sci.

[35] Mutt SJ, Hyppönen E, Saarnio J, Järvelin MR, Herzig KH, Vitamin D (2014). Adipose tissue-more than storage. Front Physiol.

[36] Shi H, Norman AW, Okamura WH, Sen A, Zemel MB (2001). 1alpha,25-Dihydroxyvitamin D3 modulates human adipocyte metabolism via nongenomic action. FASEB J.

[37] Vanlint S (2013). Vitamin D and Obesity. Nutrients.

[38] Drincic AT, Armas LA, Van Diest EE, Heaney RP (2012). Volumetric dilution, rather than sequestration best explains the low vitamin D status of obesity. Obesity (Silver Spring).

[39] Foss YJ (2009). Vitamin D deficiency is the cause of common obesity. Med Hypotheses.

[40] Wortsman J, Matsuoka LY, Chen TC, Lu Z, Holick MF (2000). Decreased bioavailability of vitamin D in obesity. Am J Clin Nutr.

[41] Zemel MB (2002). Regulation of adiposity and obesity risk by dietary calcium: mechanisms and implications. J Am Coll Nutr.

[42] Duncan RE, Ahmadian M, Jaworski K, Sarkadi-Nagy E, Sul HS (2007). Regulation of lipolysis in adipocytes. Annu Rev Nutr.

[43] Lamendola CA, Ariel D, Feldman D, Reaven GM (2012). Relations between obesity, insulin resistance, and 25-hydroxyvitamin D. Am J Clin Nutr.

[44] Cheng S, Massaro JM, Fox CS, Larson MG, Keyes MJ, McCabe EL, Robins SJ, O’Donnell CJ, Hoffmann U (2010). Adiposity, cardiometabolic risk, and vitamin D status: the Framingham heart study. Diabetes.

[45] Khan H, Kunutsor S, Franco OH, Chowdhury R (2013). Vitamin D, type 2 diabetes and other metabolic outcomes: a systematic review and meta-analysis of prospective studies. Proc Nutr Soc.

[46] Fassina G, Maragno I, Dorigo P, Contessa AR (1969). Effect of vitamin D2 on hormone-stimulated lipolysis *in vitro*. Eur J Pharmacol.

[47] Zittermann A, Frisch S, Berthold HK, Götting C, Kuhn J, Kleesiek K, Stehle P, Koertke H, Koerfer R (2009). Vitamin D supplementation enhances the beneficial effects of weight loss on cardiovascular disease risk markers. Am J Clin Nutr.

[48] Salehpour A, Shidfar F, Hosseinpanah F, Vafa M, Razaghi M, Hoshiarrad A, Gohari M (2012). Vitamin D3 and the risk of CVD in overweight and obese women: a randomised controlled trial. Br J Nutr.

[49] Pathak K, Soares MJ, Calton EK, Zhao Y, Hallett J (2014). Vitamin D supplementation and body weight status: a systematic review and meta-analysis of randomized controlled trials. Obes Rev.

[50] Holick MF, Binkley NC, Bischoff-Ferrari HA, Gordon CM, Hanley DA, Heaney RP, Murad MH, Weaver CM (2011). Endocrine society. evaluation, treatment, and prevention of vitamin D deficiency: an endocrine society clinical practice guideline. J Clin Endocrinol Metab.

[51] Holick MF, Chen TC (2008). Vitamin D deficiency: a worldwide problem with health consequences. Am J Clin Nutr.

[52] Macdonald HM (2013). Contributions of sunlight and diet to vitamin D status. Calcif Tissue Int.

[53] Muscogiuri G, Altieri B, Annweiler C, Balercia G, Pal HB, Boucher BJ, Cannell JJ, Foresta C, Grübler MR, Kotsa K, Mascitelli L, März W, Orio F, Pilz S, Tirabassi G, Colao A (2016). Vitamin D and chronic diseases: the current state of the art. Arch Toxicol.

[54] Lentjes MA, Mulligan AA, Welch AA, Bhaniani A, Luben RN, Khaw KT (2015). Contribution of cod liver oil-related nutrients (vitamins A, D, E and eicosapentaenoic acid and docosahexaenoic acid) to daily nutrient intake and their associations with plasma concentrations in the EPIC-Norfolk cohort. J Hum Nutr Diet.

[55] Smutna M, Kruzikova K, Marsalek P, Kopriva V, Svobodova Z (2009). Fish oil and cod liver as safe and healthy food supplements. Neuro Endocrinol Lett.

[56] Black LJ, Seamans KM, Cashman KD, Kiely M (2012). An updated systematic review and meta-analysis of the efficacy of vitamin D food fortification. J Nutr.

[57] O’Donnell S, Cranney A, Horsley T, Weiler HA, Atkinson SA, Hanley DA, Ooi DS, Ward L, Barrowman N (2008). Efficacy of food fortification on serum 25-hydroxyvitamin D concentrations: systematic review. Am J Clin Nutr.

[58] Tong H, Rappold AG, Diaz-Sanchez D, Steck SE, Berntsen J, Cascio WE, Devlin RB, Samet JM (2012). Omega-3 fatty acid supplementation appears to attenuate particulate air pollution-induced cardiac effects and lipid changes in healthy middle-aged adults. Environ Health Perspect.

[59] Possamai FP, Júnior SÁ, Parisotto EB, Moratelli AM, Inácio DB, Garlet TR, Dal-Pizzol F, Filho DW (2010). Antioxidant intervention compensates oxidative stress in blood of subjects exposed to emissions from a coal electric-power plant in South Brazil. Environ Toxicol Pharmacol.

[60] Jackowski SA, Alvi AZ, Mirajkar A, Imani Z, Gamalevych Y, Shaikh NA, Jackowski G (2015). Oxidation levels of north American over-the-counter n-3 (omega-3) supplements and the influence of supplement formulation and delivery form on evaluating oxidative safety. J Nutr Sci.

[61] Riedl MA, Saxon A, Diaz-Sanchez D (2009). Oral sulforaphane increases phase II antioxidant enzymes in the human upper airway. Clin Immunol.

[62] Péter S, Holguin F, Wood LG, Clougherty JE, Raederstorff D, Antal M, Weber P, Eggersdorfer M (2015). Nutritional solutions to reduce risks of negative health impacts of air pollution. Nutrients.

[63] Egner PA, Chen JG, Zarth AT, Ng DK, Wang JB, Kensler KH, Jacobson LP, Muñoz A, Johnson JL, Groopman JD, Fahey JW, Talalay P, Zhu J, Chen TY, Qian GS, Carmella SG, Hecht SS, Kensler TW (2014). Rapid and sustainable detoxication of airborne pollutants by broccoli sprout beverage: results of a randomized clinical trial in China. Cancer Prev Res (Phila).

[64] Calderón-Garcidueñas L, Villarreal-Calderon R, Valencia-Salazar G, Henríquez-Roldán C, Gutiérrez-Castrellón P, Torres-Jardón R, Osnaya-Brizuela N, Romero L, Torres-Jardón R, Solt A, Reed W (2008). Systemic inflammation, endothelial dysfunction, and activation in clinically healthy children exposed to air pollutants. Inhal Toxicol.

[65] Calderón-Garcidueñas L, Jewells V, Galaz-Montoya C, van Zundert B, Pérez-Calatayud A, Ascencio-Ferrel E, Valencia-Salazar G, Sandoval-Cano M, Carlos E, Solorio E, Acuña-Ayala H, Torres-Jardón R, D’Angiulli A (2016). Interactive and additive influences of gender, BMI and Apolipoprotein 4 on cognition in children chronically exposed to high concentrations of PM2.5 and ozone. APOE 4 females are at highest risk in Mexico City. Environ Res.

[66] Calderón-Garcidueñas L, Franco-Lira M, D’Angiulli A, Rodríguez-Díaz J, Blaurock-Busch E, Busch Y, Chao CK, Thompson C, Mukherjee PS, Torres-Jardón R, Perry G (2015). Mexico City normal weight children exposed to high concentrations of ambient PM2.5 show high blood leptin and endothelin-1, vitamin D deficiency, and food reward hormone dysregulation versus low pollution controls. Relevance for obesity and Alzheimer disease. Environ Res.

[67] Calderón-Garcidueñas L, Mora-Tiscareño A, Francolira M, Torres-Jardón R, Peña-Cruz B, Palacios-López C, Zhu H, Kong L, Mendoza-Mendoza N, Montesinoscorrea H, Romero L, Valencia-Salazar G, Kavanaugh M, Frenk S (2013). Exposure to urban air pollution and bone health in clinically healthy six-year-old children. Arh Hig Rada Toksikol.

[68] Gorham ED, Mohr SB, Garland CF, Chaplin G, Garland FC (2007). Do sunscreens increase risk of melanoma in populations residing at higher latitudes?. Ann Epidemiol.

[69] Chesnut C, Kim JI (2012). There truly no benefit with sunscreen use and basal cell carcinoma? A critical review of the literature and the application of new sunscreen labeling rules to real-world sunscreen practices. J Skin Cancer.

[70] Sánchez G, Nova J, Rodriguez-Hernandez AE, Medina RD, Solorzano-Restrepo C, Gonzalez J, Olmos M, Godfrey K, Arevalo-Rodriguez I (2016). Sun protection for preventing basal cell and squamous cell skin cancers. Cochrane Database Syst Rev.

[71] Schalka S, Steiner D, Ravelli FN, Steiner T, Terena AC, Marçon CR, Ayres EL, Addor FA, Miot HA (2014). Brazilian consensus on photoprotection. An Bras Dermatol.

[72] Matsuoka LY, Wortsman J, Hanifan N, Holick MF (1988). Chronic sunscreen use decreases circulating concentrations of 25-hydroxyvitamin D. A preliminary study. Arch Dermatol.

[73] Maipas S, Nicolopoulou-Stamati P (2015). Sun lotion chemicals as endocrine disruptors. Hormones (Athens).

[74] Gore AC, Chappell VA, Fenton SE, Flaws JA, Nadal A, Prins GS, Toppari J, Zoeller RT (2015). EDC-2: the Endocrine Society’s second scientific statement on endocrine-disrupting chemicals. Endocr Rev.

[75] Nicolopoulou-Stamati P, Hens L, Sasco AJ (2015). Cosmetics as endocrine disruptors: are they a health risk?. Rev Endocr Metab Disord.

